# May Small Airways Dysfunction (SAD) Play a Role in the Idiopathic Pulmonary Fibrosis (IPF) and May SAD Be a Therapeutic Target?

**DOI:** 10.3390/arm92050033

**Published:** 2024-09-06

**Authors:** Dariusz Ziora

**Affiliations:** 1 Department of Lung Diseases and Tuberculosis, Faculty of Medical Sciences in Zabrze, Medical University of Silesia, 41-800 Zabrze, Poland; zioradar@wp.pl; 2 Specjalistyczny Gabinet Lekarski, 42-202 Czestochowa, Poland

**Keywords:** small airways, small airways dysfunction, idiopathic pulmonary fibrosis

## Abstract

**Highlights:**

**What are the main findings?**

- In patients with idiopathic pulmonary fibrosis (IPF) small airways < 2 mm in diameter demonstrated increased thickening of their walls and distortion of their airway lumens. These airways and airway epithelial cell populations play important role in IPF development and fibrogenesis.
- The small airways dysfunction (SAD) is found in about one third of IPF patients and can significantly contribute to worsening of respiratory symptoms, increasing of mortality risk and decreasing lung function measured by spirometry and oscillometry.

**What is the implication of the main finding?**

- Hence clinicians should pay more attention to SAD in IPF patients.
- Further studies are required to find an appropriate treatment targeting small airways and to evaluate its effectiveness in IPF.studies are required to find an appropriate treatment targeting small airways and to evaluate its effectiveness in IPF.

**Abstract:**

Small airway dysfunction (SAD) is a pathological process that affects the bronchioles and non-cartilaginous airways below 2 mm in diameter. This short review presents a link between SAD and IPF. Pathomorphological changes of small airways in fibrotic lungs are discussed. Additionally, functional abnormalities related to SAD measured by spirometry and oscillometry are presented. The problem of early detection and treatment of SAD as a procedure potentially capable of mitigating fibrosis is mentioned.

## 1. Introduction

Small airway disease (SAD), or small airway dysfunction, refers to pathophysiology within bronchioles and non-cartilaginous airways 2 mm or less in diameter, usually located down to the eighth generation of airways. Small airways in healthy people contribute only 10–20% of total resistance to air flow through all lung airways [1,2]. Hence these airways are often called the “silent zone” of the lung because the pathology occurs much earlier before the clinical symptoms and/or spirometric abnormalities appear [1,2]. Previous studies have mainly focused on the role of SAD in COPD and asthma or exposure to air pollution and cigarette smoke, where small airways inflammation, their wall thickening, lumen narrowing, and/or loss in number and/or loss of alveolar attachments play a very important role [1].

Idiopathic pulmonary fibrosis (IPF) is fibrosing interstitial pneumonia of unknown etiology associated with radiological and histological features of usual interstitial pneumonia (UIP), characterized by progressive worsening of dyspnea and lung function leading to respiratory failure. The pathological progression of IPF is a dynamic process involving complex interactions among epithelial cells, mesenchymal stem cells (MSCs), fibroblasts, immune cells, and endothelial cells. Previously, IPF was considered mainly as an inflammatory disease [3]. Currently, IPF is perceived as an epithelial-driven disease, where aberrant activation of lung epithelium produces mediators of proliferation, differentiation, and migration of fibroblasts into active myofibroblasts. These myofibroblasts secrete vast amounts of extracellular matrix ECM, which subsequently leads to remodeling of the lung architecture and fibrosis [3]. So far, many investigations have focused on changes that occur mainly within the alveolar and bronchovascular interstitium, and the role of small bronchi has not been appreciated [2].

## 2. Pathomorphological Changes of Small Airways in IPF

In 1977, for the first time, Fulmer et al. [4] pointed to the role of small airways in IPF. They performed a lung biopsy in 18 patients if they met diagnostic criteria for IPF and had normal values of the FEV1/FVC ratio (forced expiratory volume in 1 s/forced vital capacity) and normal airway resistance (Raw). The most frequent morphological abnormality of small airways was peribronchiolar fibrosis, either alone or accompanied by peribronchiolar inflammation and bronchiolitis. Only one patient (5.6%) had completely normal airways. Of the total small airways evaluated, 19% had a normal diameter, 27% were dilated, and 54% were narrowed [4]. The dominant histological abnormality accompanying narrowed airways was peribronchiolar fibrosis. However, it should be underlined that patients included in Fulmer’s study [4] met IPF diagnostic criteria in force in the late 1970s. Now IPF diagnosis is based on the criteria from 2011 or 2018 as provided by the American Thoracic Society (ATS)/European Respiratory Society (ERS)/Japanese Respiratory Society (JRS)/Latin American Thoracic Society (ALAT) [5,6].

In 2010, Figueira de Mello et al. [7] demonstrated on lung biopsies that airways below 6 mm in diameter have a greater wall area in patients with usual interstitial pneumonia and nonspecific interstitial pneumonia as compared to control subjects. Furthermore, these authors reported a narrower airway lumen area for these airways as compared to donor control subjects, and using immunohistology, they revealed increased expression of MMP-9 and MMP-7 in the bronchiolar epithelium. These findings suggested that small airways may take part in events leading to lung remodeling in interstitial pneumonias. Figueira de Mello et al. [7] also found a positive correlation between MMP-7 expression and airflow limitation reflected by FEF 25–75% in patients with UIP. They observed that small airway alterations were more prominent in areas of extensive parenchymal fibrosis and in areas of traction bronchiectasis observed in the 3D high resolution CT.

Verladen et al. [8] confirmed that in IPF lungs, small airways < 2 mm in diameter on thoracic MDCT scans demonstrated increased visibility due to thickening of their airway walls and distortion of their airway lumens. In their sophisticated study, Verleden et al. [8] compared explanted lungs from patients with severe IPF (*n* = 11) who underwent lung transplantation to a cohort of unused donor lungs. Regions of lungs with even minimal fibrosis, assessed by the high resolution micro-CT coupled with histology, were associated with a 60% reduction in the number of terminal bronchioles as compared to a normal (control) lung. The minimal diameter and the minimal lumen area of these terminal bronchioles were significantly increased; furthermore, accompanying fibroblastic foci and non-encapsulated lymphoid follicles were visible. On the other hand, pre-terminal bronchioles in IPF patients characterized a significant increase of the wall area and decreased lumen circularity irrespective of whether the sample was from a region of minimal or advanced fibrosis [8].

Recently, Ikezoe et al. [9] in an elegant study applying high-resolution micro-CT 3D imaging found that in eight IPF patients without microscopic fibrosis, the numbers of conducting terminal bronchioles and respiratory transitional bronchioles were significantly reduced. In patients with end-stage IPF compared to age-matched control subjects, the terminal bronchiole airway walls, were significantly thickened in regions of the lung that had no microscopic fibrosis. Furthermore, this study demonstrated that in regions of microscopic fibrosis in IPF lungs, the remaining terminal bronchioles had thicker airway walls and the airway lumen became distorted and dilated, leading to the formation of honeycomb cysts. This study emphasizes that a reduction and fibrosis of small airways in IPF likely occurred early, even before the development of microscopic parenchymal fibrosis.

The studies mentioned above [4,7,8,9] stay in line with the experimental investigations associated with bleomycin-injured small airways in a sheep model of pulmonary fibrosis [10]. Namely, in bleomycin-infused lung segments, a significant increase in airflow resistance was observed after 7 weeks as compared to saline-infused control lung segments. Furthermore, there were significantly increased levels of inflammation, fibrosis, airway wall area thickness, and collagen deposition in the peribronchial and peribronchiolar bleomycin-infused regions of the small airways as compared to saline-infused airways. Bronchial blood vessel density was not significantly different in bleomycin- and saline-infused lung segments [10].

By means of the endobronchial optical coherence tomography (EB-OCT), it is possible to evaluate small airways in vivo [11]. EB-OCT performed bilaterally in 97 lung location sites among 12 IPF patients with an early-stage disease and 5 control subjects, enabling us to estimate the bronchiole count and measure the small airway stereology metrics for each EB-OCT imaging site. In the IPF subjects, as compared to the control subjects, there was a significant bronchiole reduction (mean 42% loss). The higher the degree of pulmonary fibrosis, the greater the loss of bronchioles. However, even in less IPF-affected sites, a reduction of the bronchiole number was observed (33% loss). Stereology metrics showed that small airways were significantly larger and more distorted/irregular in more IPF-affected sites than in less IPF-affected sites and the control subjects. Interestingly, the less IPF-affected and control airways were statistically indistinguishable in terms of stereology parameters, suggesting that bronchiolar loss precedes architectural distortion. These findings support the view that small airway disease is a feature of early IPF, providing a novel insight into its pathogenesis and potential therapeutic targets [11].

In contrast to IPF, the SAD is well known and recognized in connective vascular disease-associated interstitial lung diseases (CVD-ILDs), especially in rheumatoid arthritis and Sjogren’s syndrome, often associated with obliterative and follicular bronchiolitis [2]. In Hypersensitivity Pneumonitis (HP), the pathology of small bronchi, i.e., inflammation in the bronchioles, characterized by granuloma formation and lymphocytic infiltrates, leading to bronchial obstruction, is also described [12]. Even in fibrotic HP (fHP), the presence of small airway-centered fibrosis with or without peribronchiolar metaplasia is still a major diagnostic criteria [12]. A crucial pathological feature in fHP is the classical “bridging fibrosis” appearance of peribronchiolar metaplasia, typically “air-centered” [13]. This feature, usually affecting >50% of bronchioles, is generally a reliable pathological marker of fHP [13].

## 3. Pulmonary Function Abnormalities Due to SAD in IPF

Morphological abnormalities of small bronchi do not clearly mean that such patients may always have functional airflow disturbances. In daily clinical practice, an accurate functional assessment of small airways is a complex process, and it requires not only spirometry with the flow-volume loop parameter registration (FEF 25, FEF 50, FEF 25–75). Frequently, the evaluation requires oscillometry, resistance measurements, plethysmography, nitrogen washout, alveolar nitric oxide, helium-oxygen flow-volume curves, Cdyn, and high-resolution computed tomography (HRCT) [1,2]. Fulmer et al. [4] analyzed the final maximal expiratory flow volume (MEFV) data by means of three methods: (a) with the flow (liters/second) plotted against the observed VC; (b) with the flow (liters/second) plotted against the percent of the observed TLC; and (c) with the flow divided by the observed TLC (liters/second per liter) plotted against volume (the percent of the observed VC remaining). Ultimately, this last mentioned method, i.e., (c), was found to be most useful. Nine patients (50%) had abnormal MEFV curves with reduced flows at low lung volumes, and nine (50%) had normal to increased flows at all lung volumes [4].

Zhang X et al. [14] provided a very simple definition of SAD, based on at least two of three spirometry parameters, including maximal mid-expiratory flow (MMEF), forced expiratory flow at 50% of vital capacity (FEF 50%), and at 75% of vital capacity (FEF 75%), which needed to be less than 65% of predicted values. SAD defined according to these criteria was found in 117 (38%) out of 308 IPF patients without other pulmonary comorbidities (including COPD, emphysema, and asthma). However, in 159 patients with FEV1 ≥ 80% predicted and FEV1/FVC ≥ 0.7, only 16% of patients had SAD.

Yin et al. [15] applied the same criteria for recognition of SAD as Zhang did [14]. Their retrospective analysis consisting of 243 IPF patients demonstrated that one-third (84/243, 35%) of IPF patients had SAD. These patients had a significantly higher mortality risk as compared to non-SAD patients (HR 1.725, *p* < 0.05), and the median survival rate was significantly shortened. Lung histopathological tests in 48 cases of patients undergoing lung transplantation presented various degrees of airway lesions, of which 18 patients (37.5%) were diagnosed with SAD before lung transplantation. These subjects had a higher proportion of airway distortion and obliteration.

In contrast to effort-dependent spirometry, oscillation techniques (oscillometry) are non-invasive methods that can measure respiratory impedance, respiratory system resistance (Rrs), and respiratory system reactance (Xrs). Rrs is an index of airway caliber, and Xrs is supposed to reflect elastic and inertial properties of the respiratory system. The oscilllometry can be performed during normal breathing regardless of the effort, even in patients with severely impaired lung function [16,17]. During the procedure, Rrs (resistance) at 5 Hz (R5) and resistance at 20 Hz (R20), the difference between R5 and R20 (R5–R20), reactance at 5 Hz (X5), resonant frequency (Fres), and low frequency reactance area (ALX) are usually measured and analyzed [16,17]. Measurements are performed during the expiratory phase (Ex) and the inspiratory phase (In) of the respiratory cycle. The oscillometric parameters (R5–R20), reactance at 5 Hz, (Fres) and ALX usually significantly correlate with FEF 25–75% in asthma and COPD patients. When compared to FEF 25–75%, oscillometry parameters R5–R20, X5, and Fres had improved sensitivity to detect SAD in the nonobstructive group [16].

In 2016, Mikamo et al. [18] confirmed that oscillometry was a useful method for the detection and evaluation of SAD in patients with interstitial lung diseases (ILDs). They studied 90 patients with different ILDs. According to the HRCT findings (mosaic attenuation, air trapping, and centrilobular micronodules), 19 patients were classified as having SAD (the presence group) and 71 as not having SAD (the absence group). The mean values of pulmonary function parameters (%FVC, %FEV1, FEV1/FVC, FEF 25–75, IC, TLC, FRC, RV/TLC, and %DLCO) did not differ between the two groups. There were no differences in R5 and R20 values between the groups, whereas values of R5–R20 were significantly higher in the presence group than in the absence group. The absolute values of X5, Fres, and ALX were significantly higher in the presence group than in the absence group. A univariate analysis revealed that R5–R20, X5, Fres, and ALX were significantly associated with the presence of SAD. In addition, the analysis of ROC curves suggested that Fres and DFres were useful markers for detecting and evaluating SAD in patients with ILD. It is worth noting that Mikamo’s et al. results and conclusions differed from previous results obtained by van Nord et al. [19]. Namely van Nord et al. [19] believed that the oscillometry cannot be used in clinical practice to differentiate obstructive and restrictive changes in patients with ILD. This was especially true in patients with severe restriction (TLC less than 50% of predicted) in whom the significant increase of Rrs at low frequencies was observed.

In a prospective study performed by Hu et al. [17], the SAD in IPF patients was defined according to the oscillometry parameters (ALX > 0.44 kPa/L) at the baseline and difference R5–R20. Interestingly, parameters R5-R20, X5, and Fres demonstrated no correlation with spirometric parameters and symptom scores. Only ALX was correlated with FEV1%, FEF 25–75, and SGRQ. In 23 IPF patients without coexisting emphysema who additionally received bronchodilator treatment (LABA/LAMA, LABA/GKS, LAMA/LABA/GKS), there were significant improvements in the CAT score and the SGRQ activity domain score as compared to patients without the bronchodilator treatment (n = 18), despite no significant changes in the spirometry and IOS parameters. In IPF patients with SAD diagnosed by oscillometry, there was a significant improvement in FEV_1_, FEF 25–75%, and CAT score after the bronchodilator treatment. Hence the IOS parameters appear to be a potential guide for additional bronchodilator treatment in IPF patients with SAD. Cotin et al. [20] suggested that in IPF combined with emphysema, inhaled bronchodilators should be used if the airflow obstruction (FEV1/FVC < 0.7) is present [20]. Assayag et al. [21] noticed that 9.1% of subjects from a large cohort of 551 IPF patients had a positive bronchodilatation test (i.e., an increase in FEV1 or FVC ≥12% and ≥200 milliliters after salbutamol inhalations). They assumed that probably comorbid COPD or asthma were the main reason for a positive bronchodilatation test. In the observation study by Fulmer et al. [4], Cdyn (dynamic compliance), another sensitive test for the estimation of SAD performed in 17 patients was frequency dependent in 10 and frequency independent in 7 patients. The use of bronchodilators did not cause Cdyn to become independent of frequency, indicating fixed SAD in IPF patients without additional emphysematous changes [4]. The comparison of small airways function in IPF patients to patients suffering from COPD proved that the mean values of oscillometric parameters (R5, R20, R5–R20) were significantly higher in COPD than in IPF [22,23]. This fact suggests that small airway resistance and small airway dysfunction were more pronounced and more advanced in COPD than in IPF [22]. Also, the percentage of patients with COPD and SAD recognized by X5 exp. was significantly higher than the percentage of patients with IPF and SAD [94% vs. 18%] [23].

What about oscillometry in monitoring IPF and the progress of the disease? Interestingly and unexpectedly, airway resistance parameters (R5, R20, R5–R20) reflecting SAD did not change significantly during the 12 months’ follow-up. Only ALX rose significantly simultaneously to the decline of FVC, confirming the functional disease progress [24].

## 4. Small Airways Dysfunction as a Therapeutic Target in ILD?

Taking into account that IPF has a preclinical phase identified only histologically in which small conducting airways play a potential role in further IPF pathogenesis, it seems logical that small airways should not be forgotten as a therapeutic target [25]. Hence, maybe aerosolized drug delivery onto the airway epithelium could provide an advantageous therapeutic option in the early stage of IPF [25]. The effectiveness of the inhalation route of drug delivery in fibrotic lung was proven by Usmani et al. [26]. Aerosol particles sized 1.5 μm in diameter were able to effectively penetrate the peripheral areas of lungs in subjects with IPF [26]. Moreover, a new type jet nebulizer with breath enhancement, producing particles of the mass median aerodynamic diameter (MMAD) of circa 1 um, enabled to control drug delivery to the alveoli and small airways while avoiding deposition in the throat and central airways [27]. Using slow and deep breathing, more than a half of the emitted interferon 1 dose deposited in the peripheral lung both in healthy and IPF patients [27]. However, it seems too early to answer the crucial questions: What kind of therapeutic interventions might target small airway disease to modify the IPF outcome? Will nebulized anti-inflammatory, antifibrotic, or maybe mucolytic drugs applied in early IPF stages be effective in the inhibition of fibrosis and further lung remodeling? Will they be effective even if the number of bronchioles is reduced and anatomical changes have already occurred?

In contrast to asthma, the effectiveness of anti-inflammatory steroids administered by inhalation in IPF may be questionable because there is no evidence to support the use of even the systemic corticosteroids in IPF patients [28]. Currently, an antifibrotic drug pirfenidone administered via nebulization seems to be a promising therapeutic alternative as compared to the oral way of treatment [29]. Furthermore, numerous phase 1 or phase 2 studies focusing on the administration of various new drugs by inhalation in patients with IPF are underway [30]. For example, Silybin (a heat shock protein C-terminal inhibitor), GB0139 (a galectin-3 inhibitor), Cell Free Fat Extract (CEFFE), SM04646, TD-1058, N-Acetylcysteine (NAC), ARO-MMP7, PRS-220, and RIN-PF-302 solutions are most frequently tested [30]. However, it should be underlined that the goal of these studies is not the SAD cure or prevention but the estimation of effectiveness and tolerance of new drugs in lower doses than those administered orally.

Perhaps the ongoing study “Advancing Prevention of Pulmonary Fibrosis (APPLe)” ClinicalTrials.gov ID NCT04564183 will offer new guidelines. This study plans to determine if IPF can be detected early, possibly in the preclinical phase, before lungs are permanently scarred. This study will enroll participants who are not currently diagnosed with pulmonary fibrosis but who have family members with pulmonary fibrosis [31]. In individuals who are at risk but without visually identifiable fibrosis, applying minimally invasive techniques such as EB-OCT, high-resolution micro-CT, or hyperpolarized Xenon 129 MRI, it should be possible to elucidate the sequence of events and the evolution of small airway abnormalities in vivo [32]. Additionally, advanced genomic techniques could provide new insight on small airways involvement in IPF pathogenesis [32]. It has been established so far, that expression of the *MUC5B* gene, responsible for mucin production in the epithelium lining of respiratory bronchioles, is the strong risk factor for pulmonary fibrosis [33]. Probably the excessive mucin accumulation in small airways may lead to retention of injurious particles, resulting in focal and persistent injury, repair, and regeneration. Perhaps improving mucociliary clearance and/or prevention of mucin overproduction may be additional goals of treatment in IPF. Future studies should also consider whether *MUC5B* genotypes and expression levels influence the small airway number and morphology [32]. Recently, the influence of *MUC5B* on the small airway secretory cell migration, differentiation, and turnover has been suggested [34]. Namely, Kurche et al. [34] found that the *MUC5B* promoter variant was associated with reduced small airway secretory cells (SASC) markers but increased mucin and cilia markers. The authors hypothesized that “attrition of SASCs is a common feature of risk and development of lung fibrosis” and proposed that “the *MUC5B* variant potentially deregulates *MUC5B* silencing and locks in mucosecretory differentiation of SASC cells mobilized by injury”. Taking into account the fact that polymorphism in the promoter region of the *MUC5B* gene plays a role in IPF pathogenesis and the honeycomb areas are lined with bronchiolar-like epithelium, Tanabe et al. [35] speculated that the small airway remodeling might be involved in the honeycomb formation. Using a combination of histologic assessment and 3D microCT, they found in the IPF lung a reduction in the number of terminal bronchioles associated with honeycomb formation but not with patchy fibrosis or fibroblast foci [35]. Therefore, they assume that the honeycomb formation and fibrogenesis might be two separate processes [35]. Interestingly, single-cell RNA-Sequencing (scRNA-Seq) studies revealed a significant increase in airway ciliated and airway basal cell populations with a decrease of alveolar type 1 (AT1) and 2 (AT2) cells [36]. The basal cell expansion, seen as a common feature of epithelial remodeling in IPF, plays an important role not only in disease pathogenesis but also in disease progression [37]. In IPF, significant heterogeneity is observed among basal cells. Multipotent and secretory-primed subset basal cells (SPB) were presented in distal airways and honeycomb regions of lungs obtained from patients undergoing lung transplantation. The identification of SPB cells at sites where MUC5B is also expressed has linked these results to MUC5B allele-specific susceptibility to IPF. These observations may also suggest modulation of basal cell priming as an additional therapeutic strategy in IPF [38].

## 5. Concluding Remarks

Before 2010, only a few studies investigated the role of small airway dysfunction (SAD) in idiopathic pulmonary fibrosis (IPF). In the last decade, our understanding of IPF pathogenesis has changed, and multiple pieces of evidence strongly argue for an important role of small airways and airway epithelial cell populations in disease development and fibrogenesis. The SAD found in about one third of IPF patients can significantly contribute to decreased lung function, worsening respiratory symptoms, and mortality risk. These findings suggest that clinicians should pay more attention to SAD in IPF patients. However, further studies are required to find an appropriate treatment targeting small airways and to evaluate its effectiveness in IPF.

## Data Availability

No new data were created or analyzed in this study. Data sharing is not applicable to this article.
